# Microcalcifications, mammographic breast density, and risk of breast cancer: a cohort study

**DOI:** 10.1186/s13058-022-01594-0

**Published:** 2022-12-21

**Authors:** Soyeoun Kim, Thi Xuan Mai Tran, Huiyeon Song, Boyoung Park

**Affiliations:** 1 Department of Preventive Medicine, Hanyang University College of Medicine, Seoul, Republic of Korea; 2 Department of Epidemiology and Biostatistics, Graduate School of Public Health, Hanyang University, Seoul, Republic of Korea

**Keywords:** Mammography screening, Breast density, Microcalcification, Breast cancer, Risk factor

## Abstract

**Background:**

Breast density and microcalcifications are strongly associated with the risk of breast cancer. However, few studies have evaluated the combined association between these two factors and breast cancer risk. We investigated the association between breast density, microcalcifications, and risk of breast cancer.

**Methods:**

This cohort study included 3,910,815 women aged 40–74 years who were screened for breast cancer between 2009 and 2010 and followed up until 2020. The National Health Insurance Service database includes national health-screening results from the national breast cancer screening program, which is an organized screening program provided every 2 years for all women aged 40 years or older. Breast density was assessed based on the Breast Imaging Reporting and Data System (BI-RADS) 4^th^ edition, mostly through visual assessment by radiologists. The presence or absence of microcalcifications was obtained from the mammographic results. Cox proportional hazard regression for breast cancer risk was used to estimate hazard ratios (aHRs) adjusted for breast cancer risk factors.

**Results:**

A total of 58,315 women developed breast cancer during a median follow-up of 10.8 years. Women with breast cancer had a higher proportion of microcalcifications than women without breast cancer (0.9% vs. 0.3%). After adjusting for breast density, women with microcalcification had a 3.07-fold (95% confidence interval [CI] 2.82–3.35) increased risk of breast cancer compared to women without microcalcification. The combined association between microcalcification and breast density dramatically increased the risk of breast cancer, corresponding to a higher level of breast density. Among postmenopausal women, the highest risk group was women with BI-RADS 4 and microcalcification. These women had more than a sevenfold higher risk than women with BI-RADS 1 and non-microcalcification (aHR, 7.26; 95% CI 5.01–10.53).

**Conclusion:**

Microcalcification is an independent risk factor for breast cancer, and its risk is elevated when combined with breast density.

**Supplementary Information:**

The online version contains supplementary material available at 10.1186/s13058-022-01594-0.

## Background

Breast cancer is the most common cancer and the fifth leading cause of cancer-related deaths, ranking first for incidence in 159 countries and mortality in 110 countries [1]. A population-based mammographic breast cancer screening program is cost-effective for reducing breast cancer mortality through early detection and appropriate treatment at an early stage [1]. The World Health Organization recommends organized mammographic screening every 2 years for women aged 50–69 years with an average risk in well-resourced settings [2]. In Korea, the national breast cancer screening program provides mammography screening for women aged 40 years or older every two years [3]. Mammographic breast cancer screening reduces mortality [4, 5]. It may reveal breast features that characterize benign breast disease and those that suggest an increased breast cancer risk, such as high breast density and an aberrant texture [4–7]. Studies have suggested that breast density and structural features identified through mammography increase breast cancer independently or jointly, suggesting personalized screening strategies using routine screening information [4–7].

Microcalcifications are deposits of calcium oxalate or calcium phosphate with a diameter of < 1 mm that can be identified on mammography as small bright dots [8]. Breast microcalcifications are not breast cancers themselves but represent approximately one-third of malignant breast lesions and are a well-recognized risk factor for breast cancer [9, 10]. In addition, women with dense breasts have an increased breast cancer risk compared to women with non-dense breasts [11]. A recent study suggested that a combination of mammographic features, including density, microcalcification, and mass, enables the identification of high-risk groups [12]. Although breast density and microcalcifications are well-recognized as strong independent risk factors for breast cancer, few studies have investigated the combined association of these two factors with breast cancer risk [9].

This study aimed to investigate the independent association between microcalcification and the risk of subsequent breast cancer, considering the effect of mammographic density, by using data from a retrospective cohort of East Asian women with normal mammographic breast cancer screening results. In addition, joint associations between breast density, microcalcifications, and breast cancer risk were assessed.

## Methods

### Settings and study population

This retrospective cohort study used claims data obtained from the National Health Information Database of the National Health Insurance Service, a compulsory health insurance system covering the entire Korean population. The database includes information on demographics, healthcare utilization, vital statistics, and national health-screening results [13]. In addition, mammographic screening information was obtained from breast cancer screening program data, which were provided every two years for all women aged 40 years or older [14].

**Fig. 1:**
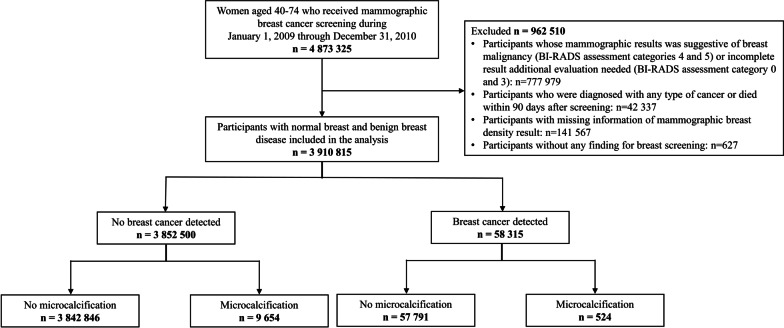
Flowchart of the selection of the eligible study population

The initial cohort included 4,873,325 women aged 40–74 who underwent breast cancer screening between 2009 and 2010 and were followed up until the date of breast cancer diagnosis, date of death, or December 31, 2020, whichever came first (Fig. 1). If participants underwent mammographic screening more than once during the study period, we used data from the first screening. The following exclusion criteria were applied to select women included in the analysis: (1) participants who were diagnosed with any cancer or died within 3 months of screening to avoid the possibility of including screening-detected cancer; (2) participants whose mammographic screening results were suspicious abnormalities, highly suggestive of malignancy, or incomplete; and (3) participants whose main exposure information was missing (breast density or microcalcification). In total, 3,910,815 women who underwent negative screening were included in the analysis.

The study was approved by the Institutional Review Board (approval no. HYUIRB-202106-003-1). The requirement for informed consent was waived because the database was constructed after anonymization of individual identities. This work was supported by a National Research Foundation of Korea grant funded by the Korean government (MSIT) (grant no. 2021R1A2C1011958). This work was partly supported by Institute of Information & Communications Technology Planning & Evaluation (IITP) grant funded by the Korea government (MSIT) (No.2020-0-01,373, Artificial Intelligence Graduate School Program (Hanyang University)) and the research fund of Hanyang University (HY- 202,100,000,670,061).

### Mammographic breast density and microcalcification

Information on mammographic breast density and microcalcification was extracted from the mammography screening results, which were read by trained radiologists at each screening center. The results of the Korean national breast cancer screening were recorded based on the BI-RADS 4th edition since 2009. Each breast’s density was assessed according to the BI-RADS 4th edition guidelines (< 25% glandular tissue, BI-RADS 1; 25–50% glandular tissue, BI-RADS 2; 50–75% glandular tissue, BI-RADS 3; > 75% glandular tissue, BI-RADS 4) and the presence and location of a mass, typically benign calcification, microcalcification, asymmetry, architectural distortion, and associated features, were recorded. Based on these findings, the final assessment of the BI-RADS category was recorded. Microcalcification was defined as a record of microcalcification in the mammography results.

### Breast cancer cases

In Korea, the national health insurance policy has set a cost-sharing rate from 0 to 10% of the total medical expenditures for patients with high medical expenses, including severe diseases such as cancer, rare diseases, and incurable diseases [15]. Thus, patients with cancer have a special payment reduction program. Incident cancer cases are registered in this system, and special codes are given to cancer patients in the NHID. The ascertainment of cancer cases in our study was obtained from the healthcare utilization database using a combination of ICD-10 codes for breast cancer and catastrophic illness codes. The primary outcome was a breast cancer event, which was defined as a combination of the International Classification of Disease-10 code of invasive breast cancer (C50) or ductal carcinoma in situ (D05), in combination with the catastrophic illness code. This definition of cancer has a sensitivity of 98.1% compared with that of the Korean Central Cancer Registry, which contains a register of 90% of cancer cases nationwide [16].

### Covariates

We considered the following variables for adjustment in the analysis: age at screening, the cut-off for BMI was applied according to the WHO Asia–Pacific recommendation, which defines overweight as BMI ≥ 23 kg/m^2^ and obesity as BMI ≥ 25 kg/m^2^ [17, 18], family history of breast cancer among first-degree relatives, number of delivered children, smoking (experience of smoking), alcohol consumption (drinking even once a week), physical activity (high-or moderate-intensity, or walking at least once a week), age at menarche, history of breastfeeding, and use of oral contraceptives. For postmenopausal women, the age at menopause, menopausal status, and history of hormone replacement therapy were also included as adjustment factors. Except for BMI, information on the above-mentioned covariates was collected using standardized questionnaires and self-reported by the participants at each screening center during health examinations and cancer screening.

### Statistical analysis

The distribution of breast cancer events in our cohort was described with respect to the presence of microcalcifications (Additional file 1: Appendix 1). Descriptive statistics of baseline characteristics at the screening examination of study participants who developed breast cancer were compared using the chi-square test or Student’s t-test. The 5-year risk of developing breast cancer was calculated according to the BI-RADS density category for those with and without microcalcification, and menopausal status among all participants. The 5-year breast cancer risk was estimated as the number of cases diagnosed with breast cancer within 5 years of screening. Additionally, the Gray test was used to identify the equality of the cumulative incidence functions between the two groups. To quantify the association between microcalcification and the risk of breast cancer, Cox proportional hazard regression (HR) analysis was used to model the time from screening to breast cancer diagnosis with adjustment for other covariates. The assumption of proportional hazards was examined using Kaplan–Meier curves, and parallel lines of the log–log survival distribution function were identified. In the Cox regression model, breast cancer events were the primary outcomes. All participants were followed up until the date of any cancer diagnosis, including breast cancer diagnosis, date of death, or December 31, 2020, which ever came first. The censored cases were those who did not develop breast cancer (including other types of cancer development or death) or were alive until December 31, 2020. To quantify the independent association between microcalcification and breast cancer risk, analyses were performed with and without adjustments for breast density together with adjustment for other covariates. The analysis was conducted on the total population and further stratified by menopausal status. Finally, to quantify the joint associations between breast density, microcalcification, and breast cancer risk, the participants were classified into a combination of BI-RADS category and the presence of microcalcification, and the HR was presented with women with BI-RADS density category 1 and no microcalcification as the reference group. In addition, we assessed the effect of microcalcification on breast cancer risk by breast density category and then assessed the significant interaction of these two factors on breast cancer risk using the extra-sum of square F test. Additionally, to evaluate the trend of breast density categories across the development of breast cancer and the comparison by the presence of microcalcification, we obtained the p-value for trend from the asymptotic test to evaluate the trend between the exposure and the outcome. All reported *p*-values were two-sided with a type I error (*α* < 0.05) and were considered statistically significant. Statistical analyses were performed using the SAS statistical software (version 9.4; SAS Institute, Cary, NC, USA).

## Results

Among the 3,910,815 women included in the analysis (Fig. 1), 58,315 were diagnosed with breast cancer during a median follow-up of 10.8 years. After screening, average time of breast cancer diagnosis was 8.2 ± 1.8 years in all women. In women with microcalcifications, the average time of breast cancer diagnosis was 7.9 ± 1.8 years, whereas in women without microcalcifications, the average time of breast cancer diagnosis was 8.5 ± 1.8 years. Baseline characteristics of the women included in this study are shown in Table 1. The mean age of the women at screening was 54.69 ± 9.6 years. The proportion of women with microcalcifications was 0.3%, and that of women with dense breasts (BI-RADS density classifications 3 and 4) was 38.4%. Women with a breast cancer development had a higher proportion of microcalcifications and dense breasts than women without a breast cancer development (0.9% vs. 0.3% for microcalcifications and 38.4% vs. 55.4% for BI-RADS breast density, respectively).

**Table 1:** Baseline characteristics at screening examination of study participants

Characteristic	No breast cancer development *n* = 3 852 500	(%)	Breast cancer development *n* = 58 315	(%)
*Age (years)*				
Mean/SD	54.07/ 9.39		51.55/ 8.53	
*BMI status (kg/m*^*2*^*)*				
< 23	1 557 014	(40.4)	24 644	(42.3)
≥ 23	2 294 644	(59.6)	33 656	(57.7)
Missing	842	(0.0)	15	(0.0)
*Age at menarche (years)*				
< 15	859 664	(22.3)	17 196	(29.5)
≥ 15	2 866 951	(74.4)	39 252	(67.3)
Missing	125 885	(3.3)	1 867	(3.2)
*Menopausal status*				
No	1 585 111	(41.1)	31 567	(54.1)
Yes	2 201 388	(57.1)	25 724	(44.1)
Missing	66 001	(1.7)	1 024	(1.8)
*Age at menopause (years)*				
Premenopausal	1 585 111	(41.1)	31 567	(54.1)
< 52	1 312 303	(34.1)	13 860	(23.8)
≥ 52	755 358	(19.6)	10 033	(17.2)
Missing	199 728	(5.2)	2 855	(4.9)
*Parity*				
Nulliparous	135 055	(3.5)	3 034	(5.2)
Parous	3 653 852	(94.8)	54 292	(93.1)
Missing	63 593	(1.7)	989	(1.7)
*Breastfeeding*				
Never	513 723	(13.3)	10 360	(17.8)
Ever	3 261 612	(84.7)	46 730	(80.1)
Missing/NA	77 165	(2.0)	1 225	(2.1)
*Oral contraceptive use*				
Never	3 047 538	(79.1)	46 300	(79.4)
Ever	735 719	(19.1)	10 952	(18.8)
Missing	69 243	(1.8)	1 063	(1.8)
*Family history of breast cancer in a first-degree relative*				
No	3 794 665	(98.5)	56 470	(96.8)
Yes	57 835	(1.5)	1 845	(3.2)
*Physical activity*				
No	1 099 679	(28.5)	15 240	(26.1)
Yes	2 719 111	(70.6)	42 607	(73.1)
Missing	33 710	(0.9)	468	(0.8)
*Smoking status*				
Never smoked	3 667 715	(95.2)	55 157	(94.6)
Ever smoked	167 886	(4.4)	2 897	(5.0)
Missing	16 899	(0.4)	261	(0.4)
*Drinking status*				
No drinking	3 096 440	(80.4)	45 263	(77.6)
Drinking	727 857	(18.9)	12 613	(21.6)
Missing	28 203	(0.7)	439	(0.8)
*Hormone replacement therapy*				
Premenopausal	1 585 111	(41.1)	31 567	(54.1)
Never	1 762 573	(45.8)	19 146	(32.8)
Ever	426 097	(11.1)	6 685	(11.5)
Missing	78 719	(2.0)	917	(1.6)
*Microcalcification*				
No	3 842 846	(99.7)	57 791	(99.1)
Yes	9 654	(0.3)	524	(0.9)
*BI-RADS breast density classification*				
1—Almost fatty	1 227 502	(31.9)	10 599	(18.2)
2—Scattered fibroglandular densities	1 144 267	(29.7)	15 421	(26.4)
3—Heterogeneously dense	1 021 547	(26.5)	20 566	(35.3)
4—Extremely dense	459 184	(11.9)	11 729	(20.1)

All variables excluding oral contraceptive were significant (*p* < 0.0001)

Overall, women with microcalcifications had a higher 5-year risk of breast cancer than those without microcalcifications in all the BI-RADS density categories (Table 2). In the total study population, the 5-year breast cancer risk was 0.60% (95% confidence interval [CI] 0.60–0.61). As the BI-RADS density category increased, the 5-year breast cancer risk increased, irrespective of the presence of microcalcifications. Overall, in women with microcalcification, the 5-year risk increased from 1.69% (95% CI 1.15–2.40) in BI-RADS 1 to 3.93% (95% CI 3.10–4.91) in BI-RADS 4. Women with microcalcification had a higher 5-year breast cancer risk, regardless of menopausal status and BI-RADS density categories. Overall, and in most categories, higher 5-year breast cancer risk were observed in premenopausal women than in postmenopausal women. However, postmenopausal women with microcalcification had a higher 5-year breast cancer risk than premenopausal women in BI-RADS density category 2, (2.11% [95% CI 1.32–3.21] vs. 2.77% [95% CI 2.09–3.60]), as well as category 4 (4.41% [95% CI 2.75–6.65] vs. 3.85% [95% CI 2.91–4.98]). Additional file 2: Appendix 2 shows the cumulative incidence over time since mammography was based on the presence/absence of microcalcifications. Overall, in all breast density groups, women with microcalcifications had a higher risk than those without microcalcifications. In BI-RADS 4, the 5-year risk of women without microcalcifications was 2.77% (95% CI 2.73–2.82) but that of women with microcalcifications was 8.39% (95% CI 7.80–9.00).

**Table 2:** 5-year breast cancer risk by the presence or absence of a microcalcification

Presence of microcalcification	Total No	BC events during total follow-up	BC events within 5-year	5-year risk (%)
**Total women**	3 910 815	58 315	23 267	0.60 (0.60–0.61)
**No microcalcification**				
Total	3 900 637	57 791	22 965	0.60 (0.59–0.61)
BI-RADS 1	1 236 398	10 548	3 983	0.33 (0.32–0.34)
BI-RADS 2	1 156 867	15 303	5 999	0.53 (0.52–0.54)
BI-RADS 3	1 038 262	20 338	8 212	0.80 (0.79–0.82)
BI-RADS 4	469 110	11 602	4 771	1.03 (1.00–1.06)
**Microcalcification**				
Total	10 178	524	302	3.01 (2.69–3.36)
BI-RADS 1	1 703	51	28	1.69 (1.15–2.40)
BI-RADS 2	2 821	118	71	2.56 (2.02–3.20)
BI-RADS 3	3 851	228	133	3.50 (2.95–4.12)
BI-RADS 4	1 803	127	70	3.93 (3.10–4.91)
**Premenopausal women**				
**No microcalcification**				
Total	1 611 923	31 268	12 429	0.78 (0.77–0.80)
BI-RADS 1	239 116	2 850	1 053	0.45 (0.42–0.48)
BI-RADS 2	399 866	6 031	2 373	0.60 (0.58–0.63)
BI-RADS 3	613 791	12 985	5 174	0.85 (0.83–0.88)
BI-RADS 4	359 150	9 402	3 829	1.08 (1.05–1.11)
**Microcalcification**				
Total	4 755	299	162	3.45 (2.96–4.00)
BI-RADS 1	402	22	12	3.04 (1.66–5.08)
BI-RADS 2	914	35	19	2.11 (1.32–3.21)
BI-RADS 3	2 101	143	80	3.86 (3.09–4.75)
BI-RADS 4	1 338	99	51	3.85 (2.91–4.98)
**Postmenopausal women**				
**No microcalcification**				
Total	2 221 819	25 505	10 129	0.47 (0.46–0.48)
BI-RADS 1	977 104	7 520	2 868	0.30 (0.29–0.31)
BI-RADS 2	733 417	8 945	3 494	0.49 (0.47–0.50)
BI-RADS 3	408 272	7 032	2 905	0.72 (0.70–0.75)
BI-RADS 4	103 026	2 008	862	0.85 (0.80–0.91)
**Microcalcification**				
Total	5 293	219	135	2.60 (2.19–3.06)
BI-RADS 1	1 282	29	16	1.28 (0.77–2.04)
BI-RADS 2	1 874	81	51	2.77 (2.09–3.60)
BI-RADS 3	1 698	81	49	2.92 (2.19–3.80)
BI-RADS 4	439	28	19	4.41 (2.75–6.65)

The associations between microcalcification and the risk of subsequent breast cancer, with and without adjustment for breast density, are presented in Table 3. In the model without adjustment for density, microcalcification was associated with a more than threefold increase in the risk of breast cancer in premenopausal and postmenopausal women. After adjusting for breast density, despite a slight decrease in the strength of the association, hazard ratios were all greater than threefold (3.09 [95% CI 2.83–3.36] in all women, 3.07 [95% CI 2.74–3.44] in premenopausal women, and 3.09 [95% CI 2.70–3.53] in postmenopausal women. Additionally, women with microcalcification were at increased risk of DCIS by fourfold (4.02 [95% CI 3.44–4.69]) and IBC risk by threefold (2.99 [95% CI 2.72–3.27]) after adjusting for breast density.

**Table 3:** Association between microcalcification and breast cancer risk, with and without adjustment for breast density with respect to breast cancer subtype

Menopausal status	BI-RADS breast density	aHR (95% CI) relative to no microcalcification	
		aHR^a^	95% CI
**Total breast cancer**			
*Total*			
	Model unadjusted for density	3.13	(3.04–3.61)
	Model adjusted for breast density	3.09	(2.83–3.36)
*Premenopausal*			
	Model unadjusted for density	3.23	(2.69–3.62)
	Model adjusted for breast density	3.07	(2.74–3.44)
*Postmenopausal*			
	Model unadjusted for density	3.41	(2.98–3.89)
	Model adjusted for breast density	3.09	(2.70–3.53)
**Invasive breast cancer (IBC)**			
*Total*			
	Model unadjusted for density	3.21	(2.92–3.51)
	Model adjusted for breast density	2.99	(2.72–3.27)
*Premenopausal*			
	Model unadjusted for density	3.08	(2.72–3.48)
	Model adjusted for breast density	2.92	(2.58–3.30)
*Postmenopausal*			
	Model unadjusted for density	3.36	(2.92–3.86)
	Model adjusted for breast density	3.06	(2.66–3.52)
**Breast cancer in situ (DCIS)**			
*Total*			
	Model unadjusted for density	4.33	(3.71–5.06)
	Model adjusted for breast density	4.02	(3.44–4.69)
*Premenopausal*			
	Model unadjusted for density	4.33	(3.55–5.27)
	Model adjusted for breast density	4.10	(3.36–4.99)
*Postmenopausal*			
	Model unadjusted for density	4.23	(3.28–5.45)
	Model adjusted for breast density	3.82	(2.97–4.93)

^a^Cox regression models were adjusted for age at first screening, obesity status, age at menarche, parity, oral contraceptive use, smoking status, breast feeding, drinking status, first-degree family history of breast cancer, and physical activity. The model for postmenopausal women was additionally adjusted for age at menopause, menopausal status, and hormone replacement therapy

The joint associations between microcalcification, breast density, and risk of breast cancer are shown in Table 4. Among women with BI-RADS density category 1, those with microcalcifications had a 3.42-fold (95% CI 2.60–4.50) increased risk of breast cancer. Compared to women with BI-RADS 1 but without microcalcification, women with BI-RADS category 4 had a 2.30-fold (95% CI 2.22–2.37) increased risk of breast cancer. Women with BI-RADS 4 breast density and microcalcification had a 6.65-fold (adjusted hazard ratio [aHR], 6.65; 95% CI 5.59–7.72). Among premenopausal women, the hazard ratios increased corresponding to a higher level of breast density, reaching a value of 5.95 (95% CI 4.87–7.28, *p* for trend = 0.004) in women with microcalcification and BI-RADS 4 compared with women with BI-RADS 1 but without microcalcification. In postmenopausal women, participants with BI-RADS 4 and microcalcification had a more than sevenfold higher risk of breast cancer (aHR, 7.26; 95% CI 5.01–10.53) compared with women with BI-RADS1 and without microcalcification. Additionally, the joint associations between microcalcification, breast density, and risk of breast cancer are shown with respect to breast cancer subtype. Women with BI-RADS 4 and microcalcification had a sixfold higher risk of invasive breast cancer (aHR, 6.27; 95% CI 5.19–7.57) than women with BI-RADS 1 and without microcalcification. However, women with BI-RADS 4 and microcalcification had an eightfold higher risk of DCIS (aHR, 8.30; 95% CI 3.95–17.47) than women with BI-RADS 1 and without microcalcification which indicates that there was an interaction effect between breast density and the presence of microcalcification on the risk of breast cancer (*p* < 0.001).

**Table 4:** Joint association between breast cancer, microcalcifications, and breast cancer risk with respect to breast cancer subtype

Microcalcification^a^	BI-RADS breast density								
	BI-RADS 1		BI-RADS 2		BI-RADS 3		BI-RADS 4		P-trend^b^
	Total population (No. of cases)	aHR (95% CI)	Total population (No. of cases)	aHR (95% CI)	Total population (No. of cases)	aHR (95% CI)	Total population (No. of cases)	aHR (95% CI)	
Total breast cancer									
Total									
No microcalcification	1 236 398 (10 548)	[Ref]	1 156 867 (15 303)	1.40 (1.37–1.44)	1 038 262 (20 338)	1.91 (1.86–1.96)	469 110 (11 602)	2.30 (2.23–2.37)	< 0.001
Microcalcification	1 703 (51)	3.42 (2.60–4.50)	2 821 (118)	4.50 (3.76–5.39)	3 851 (228)	5.89 (5.16–6.71)	1 803 (127)	6.65 (5.58–7.92)	< 0.001
Premenopausal									
No microcalcification	239 116 (2 850)	[Ref]	399 866 (6 031)	1.24 (1.19–1.30)	613 791 (12 985)	1.72 (1.65–1.79)	359 150 (9 402)	2.11 (2.02–2.21)	< 0.001
Microcalcification	402 (22)	4.61 (3.03–7.02)	914 (35)	3.16 (2.26–4.41)	2 101 (143)	5.58 (4.72–6.60)	1 338 (99)	5.95 (4.87–7.28)	0.004
Postmenopausal									
No microcalcification	977 104 (7 520)	[Ref]	733 417 (8 945)	1.46 (1.41–1.50)	408 272 (7 032)	1.97 (1.90–2.03)	103 026 (2 008)	2.20 (2.09–2.32)	< 0.001
Microcalcification	1 282 (29)	2.88 (2.00–4.15)	1 874 (81)	5.16 (4.15–6.43)	1 874 (81)	5.42 (4.36–6.75)	439 (28)	7.26 (5.01–10.53)	< 0.001
Invasive breast cancer (IBC)									
Total									
No microcalcification	1 236 398 (9 704)	[Ref]	1 156 867 (13 870)	1.39 (1.35–1.43)	1 038 262 (18 397)	1.98 (1.84–1.95)	469 110 (10 483)	2.29 (2.22–2.36)	< 0.001
Microcalcification	1 703 (46)	3.36 (2.51–4.48)	2 821 (107)	4.45 (3.68–5.39)	3 851 (199)	5.67 (4.90–6.49)	1 803 (109)	6.27 (5.19–7.57)	< 0.001
Premenopausal									
No microcalcification	239 116 (2604)	[Ref]	399 866 (5 440)	1.23 (1.17–1.29)	613 791 (11 688)	1.71 (1.64–1.79)	359 150 (8 462)	2.11 (2.01–2.21)	< 0.001
Microcalcification	402 (18)	4.15 (2.61–6.60)	914 (31)	3.08 (2.16–4.39)	2 101 (124)	5.36 (4.48–6.42)	1 338 (84)	5.61 (4.52–6.98)	0.007
Postmenopausal									
No microcalcification	977 104 (6 931)	[Ref]	733 417 (8 137)	1.45 (1.40–1.50)	408 272 (6 414)	1.97 (1.90–2.04)	103 026 (1 839)	2.20 (2.09–2.33)	< 0.001
Microcalcification	1 282 (28)	3.00 (2.07–4.36)	1 874 (74)	5.16 (4.10–6.49)	1 874 (71)	5.22 (4.13–6.59)	439 (25)	7.06 (4.77–10.46)	< 0.001
Breast cancer in situ (DCIS)									
Total									
No microcalcification	1 236 398 (2 149)	[Ref]	1 156 867 (3 538)	1.51 (1.43–1.60)	1 038 262 (4 972)	2.06 (1.95–2.17)	469 110 (2 849)	2.40 (2.25–2.55)	< 0.001
Microcalcification	1 703 (13)	4.25 (2.46–7.33)	2 821 (37)	6.57 (4.75–9.10)	3 851 (74)	8.42 (6.67–10.63)	1 803 (39)	8.57 (6.24–11.78)	< 0.001
Premenopausal									
No microcalcification	239 116 (639)	[Ref]	399 866 (1 455)	1.30 (1.18–1.43)	613 791 (3 287)	1.84 (1.68–2.01)	359 150 (2 357)	2.18 (1.99–2.39)	< 0.001
Microcalcification	402 (8)	7.37 (3.67–14.80)	914 (13)	5.12 (2.96–8.87)	2 101 (47)	7.69 (5.71–10.34)	1 338 (32)	7.84 (5.49–11.20)	0.216
Postmenopausal									
No microcalcification	977 104 (1 480)	[Ref]	733 417 (2 012)	1.61 (1.50–1.72)	408 272 (1 609)	2.14 (1.98–2.30)	103 026 (443)	2.24 (2.00–2.60)	< 0.001
Microcalcification	1 282 (5)	2.52 (1.05–6.06)	1 874 (24)	7.48 (5.00–11.21)	1 874 (24)	7.65 (5.10–11.46)	439 (7)	8.30 (3.95–17.47)	< 0.001

^a^ Cox regression models were adjusted for age at first screening, obesity status, age at menarche, parity, oral contraceptive use, smoking status, breastfeeding, drinking status, first-degree family history of breast cancer, and physical activity. The model for postmenopausal women was additionally adjusted for age at menopause, menopausal status, and hormone replacement therapy

^b^ *P* for trend: asymptotic test

## Discussion

In this large population-based cohort, we found that women with microcalcification detected during mammographic screening had an approximately threefold increased risk of breast cancer relative to women without microcalcification, regardless of menopausal status. The association between microcalcification and breast cancer risk did not change significantly after adjusting for breast density, suggesting an independent association that was not confounded by breast density. Both microcalcification and higher breast density categories increased breast cancer risk, suggesting a joint association of microcalcification and higher breast density with breast cancer risk. Women with microcalcification and BI-RADS density category 4 had an approximately sixfold higher risk of breast cancer than women without microcalcification and BI-RADS category 1.

Previous studies have shown an association between microcalcification identified during mammographic screening and an increased risk of breast cancer [9, 19], as we found in this study. Other studies suggested that as the number of microcalcification clusters increases, the risk of breast cancer and *ductal carcinoma *in situ likewise increases [8, 12, 20, 21]. When subjects were stratified by menopausal status, Azam et al*.* showed that the presence of three or more microcalcification clusters had a higher association with premenopausal breast cancer risk than postmenopausal breast cancer risk [9]. In this study, the overall association between microcalcification and breast cancer risk was observed in both premenopausal and postmenopausal women, with similar HRs in both groups. However, in women with BI-RADS density category 1, the association between microcalcification and breast cancer risk was stronger in premenopausal women than in postmenopausal women. In women with BI-RADS density categories 2–4, the association of microcalcification was higher in postmenopausal women than in premenopausal women or similar in both groups.

Microcalcification is more prevalent in older patients [9] and is affected by epithelial-mesenchymal transition [22], cell necrosis, and debris [19]. A previous study showed that age, breast density, genetic predictors of breast cancer, having more than two children, and long breastfeeding periods are associated with an increased risk of microcalcification clusters [9]. Other studies have revealed that body mass index, smoking, and alcohol consumption could be negatively associated with microcalcification clusters because of the protective effect of estrogen [23, 24]. Although breast density and breast microcalcification are independently associated with breast cancer, the relationship between these two factors and age is the opposite. While breast density decreases with increasing age [25], the number of microcalcification clusters increases with age increases [9]. The age-dependent prevalence of the microcalcification clusters could be explained by the effect of normal aging due to epithelial-mesenchymal transition over age [26]. A recent Swedish population study indicated that microcalcification clusters can be observed as a complementary risk indicator of breast cancer [20], whereas mammographic density is a strong risk factor for breast cancer [27].

It is unclear whether microcalcification is a risk factor for benign breast diseases, breast cancer, or an early detection marker for these diseases [28]. Microcalcification is not only a risk factor for breast cancer, but also a potential surrogate for breast cancer recurrence [32] and a prognostic marker for residual disease after excision [29]. Microcalcification can be used to indicate where breast cancer develops and when it appears [19]. However, in this study, by excluding those with mammographic screening results of suspected breast cancer cases or incomplete and developed breast cancer within three months of screening, microcalcifications with malignancy potential (surrogate marker of breast cancer) could be excluded at baseline. Thus, the results of this study suggest that microcalcifications, but not microcalcifications with malignant potential, are a risk factor for breast cancer.

Our study had several limitations, mainly because the definition of microcalcification was solely based on mammography results as information regarding biopsy results was unavailable in the National Health Insurance Service database. Despite the absence of biopsy results to explain the pathological information in detail, our results suggest a normal screening process that can be directly applied in screening settings. In the Korean National Breast Cancer Screening setting, it is not mandatory for at least two radiologists to interpret screening mammograms according to European guidelines [30]. Therefore, the breast density, presence of microcalcification, and BI-RADS classification were read by a single radiologist at each screening center. Although the BI-RADS classification has been extensively assessed, the results may vary depending on the skill and ability of the radiologist. However, in Korea, there is a standardized mammography education program that can increase the quality of interpretation [31]. In addition, inter-radiologist variability was assessed in randomly selected films from the Korean National Breast Cancer Screening Program, and the results showed an inter-radiologist variability of 0.83, which suggests high agreement in performance [32]. Another point should be noted that in proportion of women with family history of breast cancer in our study is relative lower than reported among western population [1]. A previous study on women from the western countries showed that approximately 15% of the women had a family history of breast cancer within their first-degree relatives [33]. Differences in the proportion of family history of breast cancer in women from the West and Korea might be related to different baseline breast cancer incidences in the population by race (lower breast cancer incidence in women from Asia compared with women from the West) [1]. A study conducted in women from Korea showed a similar proportion of family history of breast cancer as our result [34].

Despite these limitations, this study included approximately 4 million women from a population-based national breast cancer screening, which represents the demographic composition of women in Korea and other East Asian populations. Another point worth mentioning is that even though the association between microcalcification and increased breast cancer risk has been studied in published works [9, 19, 27], few studies have considered the combination of microcalcification, breast density, and breast cancer risk with additional stratification by menopausal status. To the best of our knowledge, this study is the largest cohort study to provide evidence of an increased risk of breast cancer in women with microcalcifications, with additional consideration of breast density. Hence, this large national cohort study with prospective ascertainment of breast cancer cases provides new evidence for the combined association of microcalcification and breast density at the population level, advancing existing interventions for the early detection of cancer among women with microcalcification and dense breasts who have a higher breast cancer risk.

## Conclusions

We found that microcalcification was an independent risk factor for breast cancer and that the risk increased when combined with breast density. To the best of our knowledge, our study is the first comprehensive study to use national breast cancer screening to study microcalcification and shed light on the risk of breast cancer associated with the presence of microcalcification.

## Supplementary Information

**Additional file 1: **Histogram of the time distribution from study enrollment (date of mammographic breast cancer screening) to breast cancer diagnosis in incident breast cancer cases. **A** Total breast cancer. **B** Total breast cancer cases without microcalcifications. **C** Total breast cancer cases with microcalcifications.

**Additional file 2: **Cumulative incidence over time since mammography was based on the presence/absence of microcalcifications and mammographic breast density. P-values were calculated using the Gray test. **A** BI-RADS 1; **B** BI-RADS 2; **C** BI-RADS 3; **D** BI-RADS 4. BI-RADS: Breast Imaging Reporting and Data System.

## Data Availability

The data that support the findings of this study are available from the NHIS, but restrictions apply to the availability of these data, which were used under license for the current study, and so are not publicly available. However, the data are available from the authors upon reasonable request and with permission from the NHIS.

## Abbreviations

BI-RADS: The breast imaging reporting and data systems
aHRs: Adjusted hazard ratios
CI: Confidence interval
HR: The hazard ratio
BMI: Body mass index
SD: Standard deviation
NA: Not applicable
NO: Number
