# Supplementary figures and images for: Microcalcifications, mammographic breast density, and risk of breast cancer: a cohort study
Source: Breast Cancer Res. 2022 Dec 21;24:96. doi: 10.1186/s13058-022-01594-0 (PMC9773568; doi:10.1186/s13058-022-01594-0)

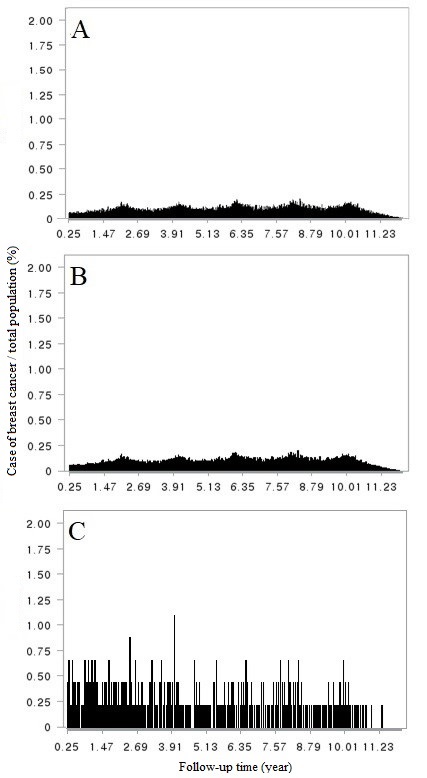

Supplement: Supplementary file 1 — Additional file 1: Histogram of the time distribution from study enrollment (date of mammographic breast cancer screening) to breast cancer diagnosis in incident breast cancer cases. A Total breast cancer. B Total breast cancer cases without microcalcifications. C Total breast cancer cases with microcalcifications. [file 13058_2022_1594_MOESM1_ESM.jpg]

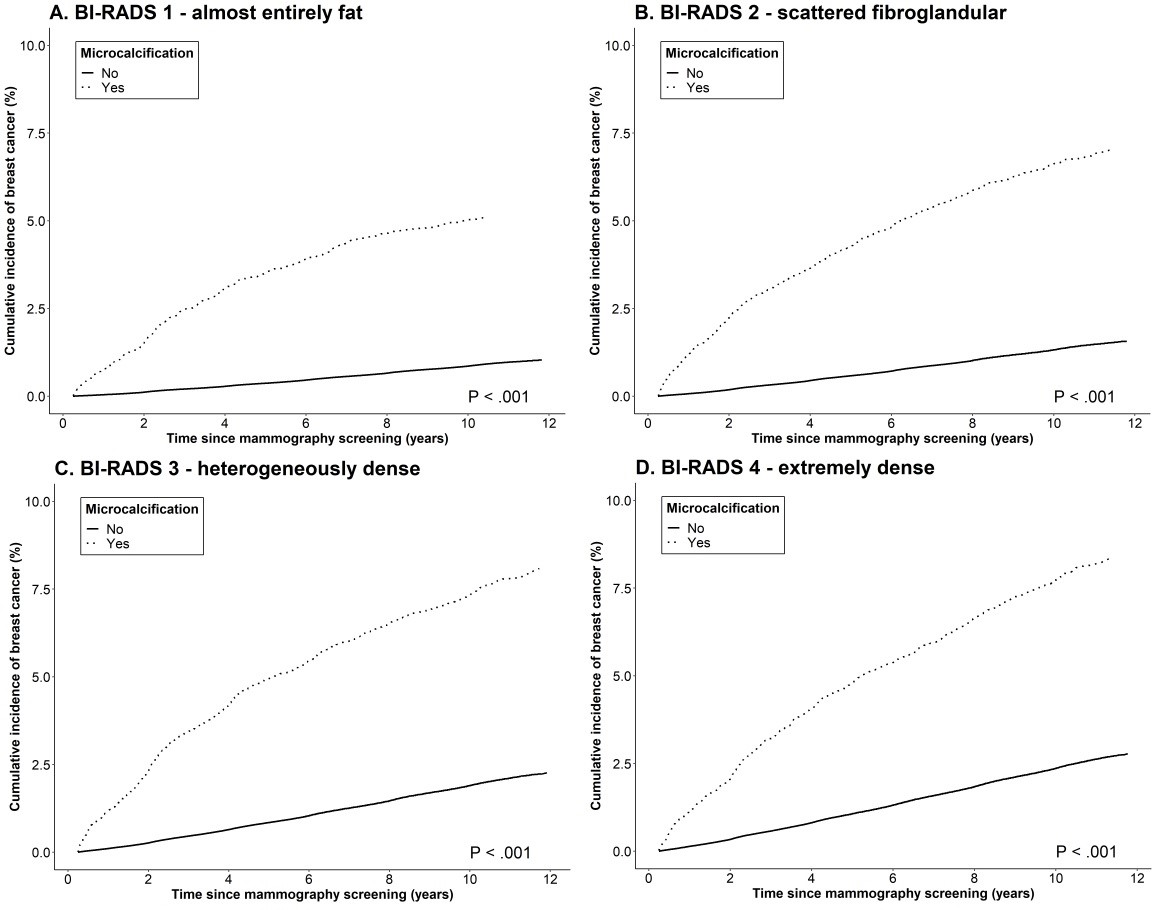

Supplement: Supplementary file 2 — Additional file 2: Cumulative incidence over time since mammography was based on the presence/absence of microcalcifications and mammographic breast density. P-values were calculated using the Gray test. A BI-RADS 1; B BI-RADS 2; C BI-RADS 3; D BI-RADS 4. BI-RADS: Breast Imaging Reporting and Data System. [file 13058_2022_1594_MOESM2_ESM.jpg]
